# Safety of direct oral anticoagulants reversal agents in older patients: an analysis of individual case safety reports of adverse drug reaction from VigiBase^®^

**DOI:** 10.1007/s40520-025-03025-4

**Published:** 2025-04-07

**Authors:** Giada Crescioli, Niccolò Lombardi, Elena Arzenton, Nicoletta Luxi, Stefano Fumagalli, Roberto Bonaiuti, Costanza Cacini, Guido Mannaioni, Gianluca Trifirò, Ugo Moretti, Alfredo Vannacci

**Affiliations:** 1 Department of Neurosciences, Psychology, Drug Research and Child Health, Section of Pharmacology and Toxicology, University of Florence, Viale G. Pieraccini, 6, 50139 Florence, Italy; 2 Tuscan Regional Centre of Pharmacovigilance, Florence, Italy; 3 Section of Pharmacology, Department of Diagnostics and Public Health, University of Verona, Verona, Italy; 4 Department of Experimental and Clinical Medicine, University of Florence, Florence, Italy; 5 Toxicology Unit, Poison Control Center, Careggi University Hospital, Florence, Italy

**Keywords:** Adverse drug reaction, Idarucizumab, Andexanet Alfa, Observational study, Pharmacovigilance.

## Abstract

**Background:**

Real-world data on adverse drug reactions (ADRs) associated with idarucizumab and andexanet alfa are limited.

**Aim:**

This study aimed to assess the frequency, the characteristics and clinical and demographic factors associated with ADRs related to their use.

**Methods:**

This is a retrospective analysis of ADR reports collected in Vigibase^®^ until May 31, 2023. Multivariable logistic regression estimated reporting odds ratios (RORs) for serious ADRs, death, and thromboembolic events according to demographical and clinical covariates.

**Results:**

A total of 1095 Individual Case Safety Reports (ICSRs) reporting idarucizumab (72%) or andexanet alfa (28%) as suspected/interacting agents were collected. Most of the subjects were males (44.5%), with a median age of 78 years, and exposed to only one suspected/interacting medication (73.6%). ADRs were defined as serious in 88.6% of cases, with a total of 614 (56.1%) fatal cases. Compared to patients without concomitant medications, probability of serious ADRs and death were both higher in those receiving ≥ 5 concomitant medications in the idarucizumab subgroup (ROR 4.04 and 1.66, respectively) and in those receiving 1–4 concomitant medications in the andexanet alfa subgroup (ROR 5.66 and 4.80, respectively). Moreover, the probability of thromboembolic events was significantly lower for subjects aged > 75 years (ROR for 75–84 years 0.55; ROR for ≥ 85 years 0.50).

**Discussion:**

In real-world, ADRs associated with idarucizumab and andexanet alfa use are generally serious, resulting in death in a high percentage of subjects.

**Conclusion:**

Clinicians should pay particular attention when managing individuals needing these drugs, especially if vulnerable and requiring polytherapy.

**Supplementary Information:**

The online version contains supplementary material available at 10.1007/s40520-025-03025-4.

## Introduction

In recent years, direct oral anticoagulants (DOACs) have been authorized for preventing stroke in individuals with non-valvular atrial fibrillation (AF), as well as for treatment of venous thromboembolism [1, 2]. These drugs act as thrombin- (dabigatran) or Factor Xa (apixaban, rivaroxaban and edoxaban) antagonists. DOACs show a favourable benefit-risk profile compared with vitamin K antagonists (VKA). Nevertheless, bleeding remains a relevant side effect and represents a concern for both clinicians and patients, since it is potentially associated with significant morbidity and mortality [3], thus contributing to undertreatment of older individuals in the *real-world* [4]. In case of bleeding caused by VKAs, the anticoagulant effect in emergency situations is reversed with a combination of vitamin K, fresh frozen plasma, prothrombin complex concentrate, or recombinant Factor VIIa [5]. For many years, a specific antidote for dabigatran and the other DOACs was not available, preventing their use in many patients [6].

In 2015, both the U.S Food and Drug Administration (FDA) and the European Medicines Agency (EMA) approved idarucizumab (Praxbind^®^, pharmaceutical company Boehringer Ingelheim, Germany), a specific reversal agent for dabigatran. Idarucizumab is a humanised monoclonal antibody fragment (Fab) that rapidly and specifically binds to and leads to sustained neutralisation (up to 24 h) and elimination of dabigatran, with an affinity that is 350 times as high as that observed with thrombin [7–9]. Its therapeutic indications comprehend surgery for emergency conditions, urgent procedures and life-threatening or uncontrolled bleeding [10]. Three years later, andexanet al.fa was approved (AndexXa^®^ in USA and Ondexxya^®^ in Europe, pharmaceutical company AstraZeneca, United Kingdom) as a reversal agent for patients treated with apixaban or rivaroxaban, in the case of life-threatening or uncontrolled bleeding [11]. Andexanet al.fa is a recombinant modified factor Xa decoy protein that exhibits high-affinity binding to the active site of factor Xa antagonists, effectively interfering with their activity, while leaving the intrinsic coagulation system unaffected. Notably, andexanet al.fa demonstrates a rapid onset of action, typically within 2 to 5 min, and maintains a half-life of approximately 60 min [12].

Several studies investigated the efficacy and tolerability of idarucizumab and andexanet al.fa. The results of RE-VERSE AD trial showed that idarucizumab was associated with a reversed anticoagulation, fast and complete in more than 98% of cases [8, 13]. Authors reported serious adverse drug events (ADEs) in 23.3% of patients. Among these, three individuals experienced a potential hypersensitivity reaction within 5 days from drug administration, and thrombotic events were reported in 4.8% of cases [13].

The ANNEXA-4 trial [14], an open-label study involving 352 DOACs users experiencing major bleeding, found that, after andexanet al.fa administration, 82% of patients achieved haemostasis restoration within 12 h, with a median reduction in anti-factor Xa activity of 92% within 18 h. However, during 30 days of follow-up, 10.4% of patients experienced thromboembolic events, and overall mortality rate was 15.7%. The most frequently reported ADEs were urinary tract infections (5%) and infusion-related reactions (3%) [15, 16].

So far, few *real-word* studies have been conducted to assess a complete safety profile of idarucizumab and andexanet al.fa in *real-world* populations. *Real-world* studies use data relating to patient health status and/or the delivery of health care routinely collected from a variety of sources. Examples of *real-world* data sources include electronic health records, medical claims, product or disease registries, data gathered from digital health technologies, and pharmacovigilance systems [17, 18]. Most available studies regarding idarucizumab and andexanet al.fa are single- or multicentre observational studies [19, 20], and none have specifically focused on adverse drug reactions (ADRs) collected through pharmacovigilance data. This calls for new analysis able to provide more details on the relationship between idarucizumab and andexanet al.fa use and safety clinical outcomes. In this frame, the aims of this observational study were to investigate the frequency of suspected ADRs associated with idarucizumab and andexanet al.fa use, as recorded in the World Health Organization (WHO) global pharmacovigilance database of individual case safety reports (ICSRs) VigiBase^®^, to describe the related subjects’ demographic and clinical characteristics, and to estimate the probability of serious ADRs, death and thromboembolic events.

## Methods

### Study design and data sources

In this retrospective observational analysis, we examined pharmacovigilance ICSRs indicating suspected ADRs associated with idarucizumab or andexanet al.fa collected until May 31, 2023 in VigiBase^®^. VigiBase^®^ was established by the World Health Organization (WHO) and is maintained by the WHO-Uppsala Monitoring Centre (WHO-UMC), gathering pharmacovigilance ICSRs of suspected ADRs from over 170 national pharmacovigilance centres participating in the WHO Programme for International Drug Monitoring [21].

### Data management

VigiLyze, a tool provided by UMC, was used for data extraction (https://who-umc.org/pv-products/vigilyze/*).* VigiBase^®^ was queried for de-duplicated ICSRs involving idarucizumab or andexanet alfa as suspected/interacting medications, identified by their Anatomical Therapeutic Chemical (ATC) classification system codes V03AB37 and V03AB38. ICSRs indicating these medications as concomitant agents were excluded. Data were merged and included in a single database created ad hoc for this analysis, using STATA v17 (StataCorp, USA).

### Demographic and clinical data

From each ICSRs the following demographic and clinical data were retrieved: country of origin, subject’s age, and sex. Suspected/interacting and concomitant medications were classified according to the ATC classification system [22, 23]. Variables such as the total number of suspected/interacting medications and concomitant medications were also analysed based on the information provided in each ICSR.

### ADR coding and outcome

ADRs were coded using the Medical Dictionary for Regulatory Activities (MedDRA) and reported as System Organ Class (SOC) and Preferred Term (PT) [24, 25]. ADRs seriousness, defined according to the WHO classification (i.e., events leading to death; life-threatening; requiring hospitalization; leading to significant disability; resulting in a congenital anomaly/birth defect; requiring other medically important condition), as well as ADRs outcome (i.e., resolution with sequelae; still unresolved; complete resolution; improvement; death; not available) were also retrieved [26].

### Statistical analysis

Descriptive statistics summarized the data. Categorical data were reported as frequencies and percentages, whereas continuous data were reported as mean and standard deviation (SD) or median values with interquartile ranges (IQRs). Multivariable logistic regression analysis models were applied to estimate the reporting odds ratios (RORs) with 95% confidence intervals (CIs) of serious ADR, death and thromboembolic events, adjusting for age, sex, number of suspected/interacting drugs, and number of concomitant medications. Statistical significance was indicated by a two-tailed *p-value* < 0.05. All analyses were performed using STATA v17 software (StataCorp, USA).

## Results

**Fig. 1:**
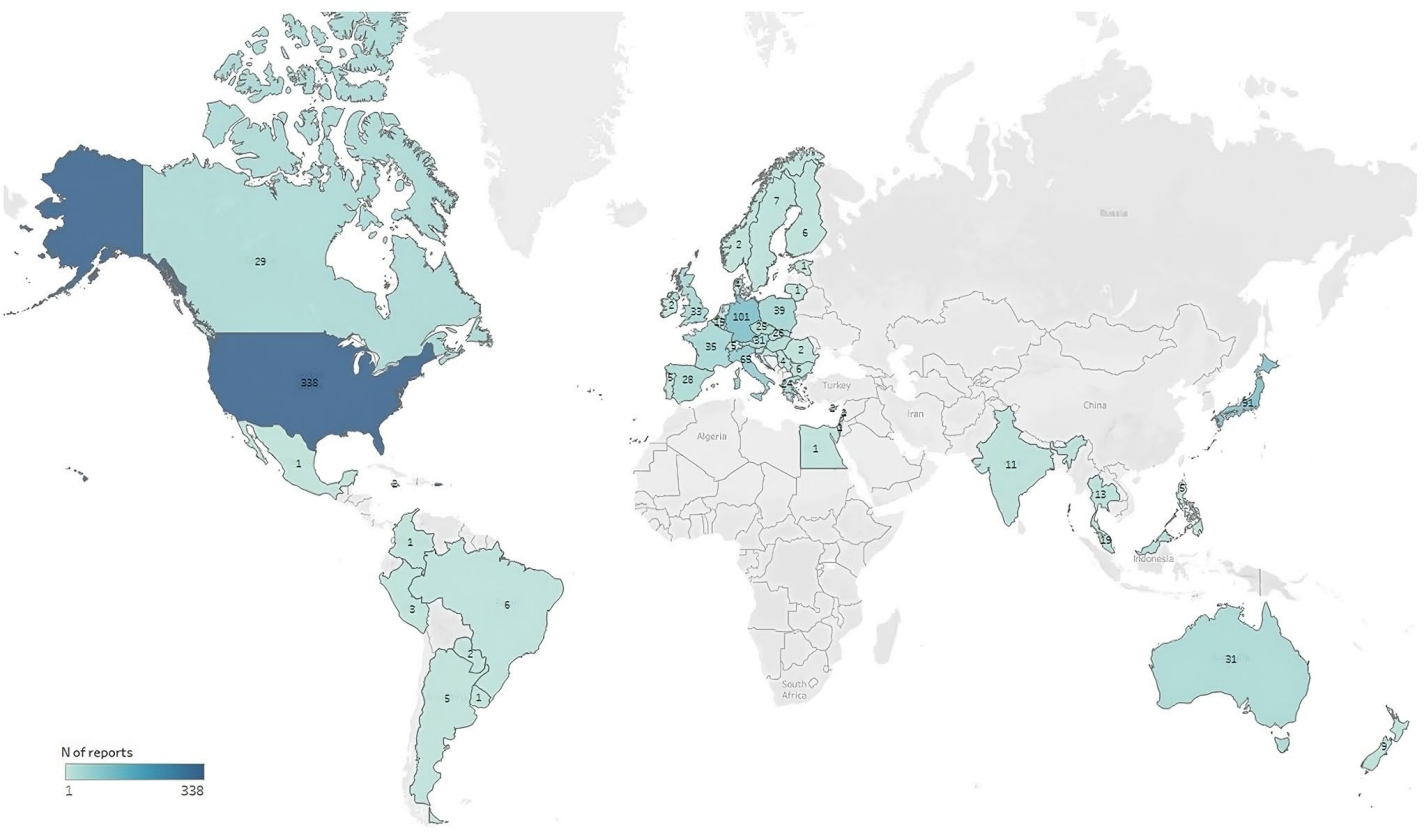
Country of origin of ICSRs with idarucizumab or andexanet alfa as suspected/interacting drug

Up to May 2023, a total of 1095 de-duplicated ICSRs reporting idarucizumab (72%) or andexanet alfa (28%) as suspected/interacting drugs were collected. Figure 1 shows the distribution of obtained ICSRs according to the Country of origin. Most subjects were males (44.5%), with a median age of 78 years, and exposed to only one suspected/interacting medication (73.6%). Subjects exposed to more than 5 concomitant drugs accounted for 18.3%. Suspected ADRs were defined as serious in 88.6% of cases (*n* = 970), with a total of 614 (56.1%) fatal cases (Table 1). Out of the total 2180 reported preferred terms, thromboembolic events accounted for 6.5%. Within this group, the most frequently were ischemic stroke (*n* = 34, 1.6%), cerebral infarction (*n* = 30, 1.4%), myocardial infarction (*n* = 46, 2.1%, of which 29 defined as acute), pulmonary embolism (*n* = 27, 1.7%), coagulopathy (*n* = 16, 0.7%), deep vein thrombosis (*n* = 16, 0.7%), unspecified thrombosis (*n* = 14, 0.6%), embolism (*n* = 10, 0.5%), and disseminated intravascular coagulation (*n* = 7, 0.3%) (*data not shown*). Supplementary Table 1 shows the most frequently reported PTs.

**Table 1:** Demographical and clinical characteristics of ICSRs with Idarucizumab and Andexanet Alfa as suspected/interacting drug

	Total sample *N* = 1095 (%)
**Suspected/interacting medication**	
Andexanet alfa	306 (27.95)
Idarucizumab	789 (72.05)
**Age (years)**	
Mean ± SD	75.2 ± 15.5
Median (IQR)	78.5 (71–84)
**Age classes**	
Adult (18–65 years)	94 (8.58)
Youngest-old (65–74 years)	160 (14.61)
Middle-old (75–84 years)	298 (27.21)
Oldest-old (≥ 85 years)	180 (16.44)
NR	363 (33.15)
**Sex**	
Males	487 (44.47)
Females^§^	384 (35.07)
NR	224 (20.46)
**Number of suspected/interacting medications**	
Mean ± SD	1.39 ± 0.89
Median (IQR)	1 (1–2)
1	806 (73.61)
2–4	274 (25.02)
> 5	15 (1.37)
**Number of concomitant medications**	
Mean ± SD	2.17 ± 4.59
Median (IQR)	0 (0–2)
0	789 (72.05)
1–4	106 (9.68)
≥ 5	200 (18.26)
**Seriousness**	
No	109 (9.95)
NR	16 (1.46)
Yes	970 (88.58)
*Death*	598 (61.65)*
*Other medically important conditions*	146 (15.05)*
*Caused or prolonged hospitalisation*	111 (11.44)*
*Life threatening*	23 (2.37)*
*Disabling/incapacitating*	10 (1.03)*
*Mixed definitions*	82 (8.45)*
Total of fatal ADRs	*614 (56.07)*

*Percentages out of 970 ADR reports defined as serious

§No cases of ADR in pregnant women

IQR: interquartile range; NR: not reported; SD: standard deviation

**Fig. 2:**
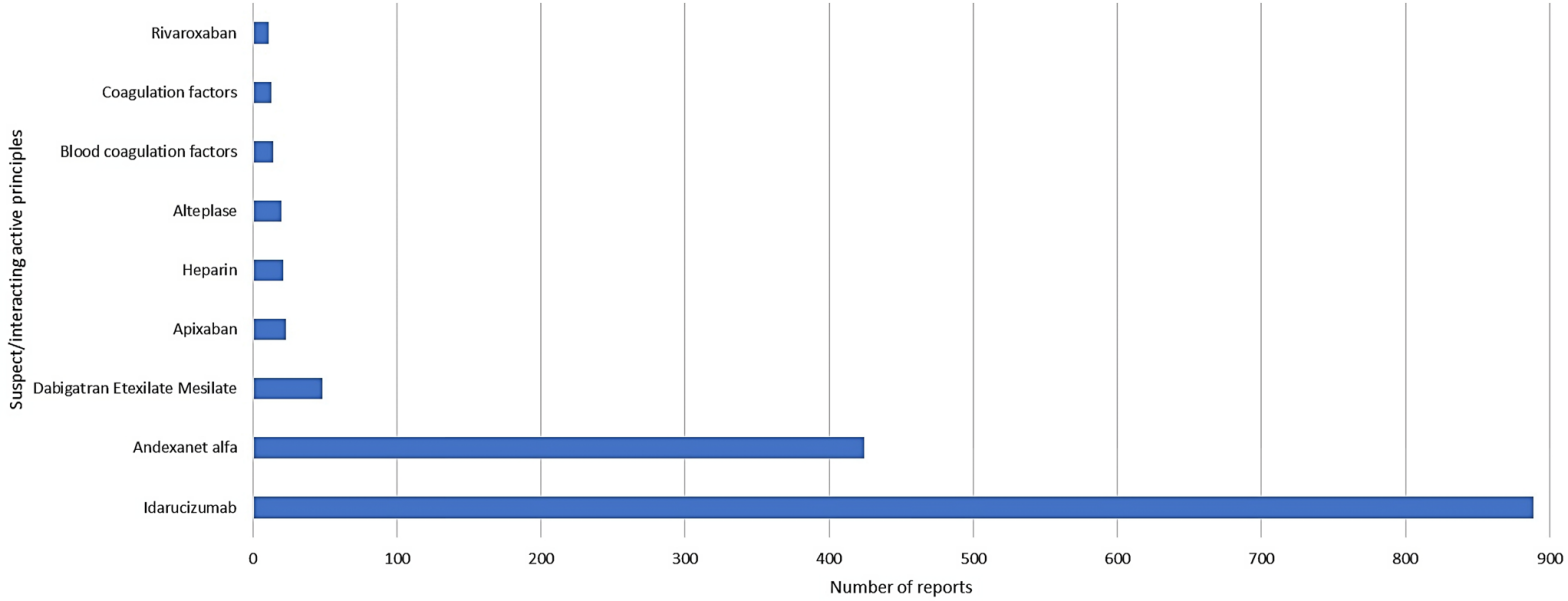
Co-reported suspected/interacting drugs in ICSRs with idarucizumab and andexanet alfa as suspected/interacting drug

Along with idarucizumab or andexanet alfa, the most frequently reported suspected/interacting medications were dabigatran (48 cases, 1.2%), apixaban (23 cases, 0.6%), heparin (21 cases, 0.5%), and alteplase (20 cases, 0.5%) (Fig. 2).

Supplementary Table 2 shows the most frequently reported concomitant medications. Over 2387 cases, 3.3% of cases reported furosemide (*n* = 79) and apixaban (*n* = 78), 3% reported bisoprolol (*n* = 72), 2.4% atorvastatin (*n* = 58), 2.1% rivaroxaban (*n* = 52), 2.1% pantoprazole (*n* = 50), and 2.0% metoprolol (*n* = 48).

Table 2 reports the estimate of regression models for the outcomes probability of serious ADR, death and thromboembolic events. In the whole population, according to the analysis, probability of serious ADR was significantly higher for individuals exposed to 1–4 and ≥ 5 concomitant medications (ROR for 1–4 concomitant medications: 2.51, 95% CI: 1.38–4.57; ROR for ≥ 5 concomitant medications: 6.35, 95% CI: 2.28–17.68). When separately analysing patients for the drug used, compared to those without reported concomitant agents, a higher probability of serious ADR was observed in subjects exposed to idarucizumab and ≥ 5 concomitant medications (ROR: 4.04, 95% CI: 1.43–11.44), and to andexanet alfa and 1–4 concomitant medications (ROR: 5.66, 95% CI: 2.11–15.24). No age-associated relation was found.

**Table 2:** Risk of serious ADR, death and thromboembolic events

	Risk of serious ADR Adjusted ROR (95% CI)			Risk of death Adjusted ROR (95% CI)			Risk of thromboembolic ADR Adjusted ROR (95% CI)		
	Overall	Idarucizumab	Andexanet alfa	Overall	Idarucizumab	Andexanet alpha	Overall	Idarucizumab	Andexanet alpha
**Overall**									
**Sex**									
Males	1	1	1	1	1	1	1	1	1
Females	0.92 (0.54–1.55)	0.98 (0.55–1.72)	0.80 (0.17–3.84)	1.00 (0.76–1.31)	1.03 (0.76–1.40)	1.01 (0.48–2.10)	1.17 (0.83–1.65)	1.15 (0.75–1.75)	1.07 (0.51–2.23)
**Age**									
Adult (18–65 years)	1	1	1	1	1	1	1	1	1
Youngest-old (65–74 years)	0.93 (0.26–3.30)	0.72 (0.18–2.89)	-	0.80 (0.47–1.34)	1.06 (0.60–1.89)	0.21 (0.06–0.77)	0.69 (0.39–1.23)	0.62 (0.30–1.28)	1.01 (0.30–3.42)
Middle-old (75–84 years)	0.67 (0.22–2.05)	0.55 (0.16–1.93)	0.40 (0.03–4.52)	0.93 (0.58–1.49)	1.10 (0.65–1.85)	0.33 (0.10–1.07)	0.55 (0.32–0.94)	0.55 (0.29–1.05)	0.76 (0.24–2.39)
Oldest-old (≥ 85 years)	0.53 (0.17–1.65)	0.51 (0.14–1.89)	0.13 (0.02–1.46)	1.13 (0.68–1.89)	1.27 (0.72–2.25)	0.60 (0.17–2.09)	0.50 (0.28–0.90)	0.54 (0.26–1.11)	0.39 (0.11–1.32)
**Number of suspected/interacting medications**									
1	1	1	1	1	1	1	1	1	1
2–4	1.36 (0.79–2.32)	1.07 (0.52–2.20)	1.04 (0.42–2.56)	0.91 (0.68–1.20)	0.63 (0.44–0.92)	1.74 (1.06–2.86)	1.30 (0.92–1.82)	1.26 (0.76–2.09)	0.67 (0.39–1.15)
≥ 5	-	-	-	0.34 (0.11-1.00)	0.77 (0.17–3.50)	0.21 (0.02–1.82)	2.10 (0.70–6.29)	1.09 (0.13–9.29)	0.72 (0.16–3.28)
**Number of concomitant medications**									
0	1	1	1	1	1	1	1	1	1
1–4	2.51 (1.38–4.57)	1.27 (0.59–2.70)	5.66 (2.11–15.24)	1.91 (1.40–2.60)	1.15 (0.76–1.71)	4.80 (2.77–8.34)	0.94 (0.65–1.38)	0.99 (0.57–1.72)	0.62 (0.35–1.11)
≥ 5	6.35 (2.28–17.68)	4.04 (1.43–11.44)	-	1.70 (1.22–2.36)	1.66 (1.13–2.44)	1.89 (0.93–3.86)	1.05 (0.71–1.56)	0.84 (0.49–1.44)	1.71 (0.82–3.57)

CI: confidence interval; ROR: Reporting Odds Ratio

Analysis was adjusted for age, sex, number of suspected/interacting drugs, and number of concomitant medications

Probability of death was significantly higher for subjects exposed to andexanet alfa and to 2–4 suspected/interacting medications compared to those exposed to only one suspected/interacting agent (ROR 1.74, 95% CI 1.06–2.86). The probability of death was also significantly higher for individuals exposed to 1–4 and ≥ 5 concomitant medications overall (ROR for 1–4 concomitant medications: 1.91, 95% CI 1.40–2.60; ROR for ≥ 5 concomitant medications: 1.70, 95% CI 1.22–2.36). Moreover, a higher probability was observed in subjects exposed to idarucizumab (ROR for ≥ 5 concomitant medications: 1.66, 95% CI 1.13–2.44) and andexanet alfa (ROR for 1–4 concomitant medications: 4.80, 95% CI 2.77–8.34) compared to those without reported concomitant agents. A significantly lower probability of death was found for individuals treated with idarucizumab and 2–4 concomitant medications (ROR 0.63, 95% CI 0.44–0.92), and for individuals treated with andexanet alfa aged 65–74 years (ROR 0.21, 95% CI 0.06–0.77) (Table 2).

In the whole population, probability of thromboembolic events was significantly lower for subjects aged 75–84 years (ROR 0.55, 95& CI 0.32–0.94), and for those aged ≥ 85 years (ROR 0.50, 95% CI 0.28–0.90) (Table 2).

## Discussion

The findings of this study reveal compelling insights into the efficacy and tolerability of idarucizumab and andexanet alfa. ADRs occurred predominantly in older patients, most of them were defined as serious, and in more than half of the sample the reported outcome was the patient’s death. Moreover, the administration of multiple concomitant medications notably heightened the probability of serious ADRs and mortality within the patient cohort.

As mentioned above, the majority of studies assessing the safety of idarucizumab and andexanet alfa in *real-world* populations are represented by single or multicentre observational studies.

A nation-wide observational cohort study performed in Italy evaluated the efficacy and safety of thrombolysis preceded by dabigatran reversal with idarucizumab in people with acute ischemic stroke [27]. Symptomatic intracranial haemorrhage (sICH) and death occurred in 10.3% (4/39) and 17.9% (7/39) of patients treated with idarucizumab. Moreover, the systematic review performed by the same authors found that thrombolysis after dabigatran reversal carried a non-significant trend for increased risk of sICH (OR = 1.53), and death (OR = 1.53). Another recent systematic review and meta-analysis of clinical trials, cohort studies, case-control studies, cross-sectional studies and case series published in 2023 [28] showed that idarucizumab use was associated with a pooled incidence of all-cause mortality and thromboembolic events at any follow-up duration of 13.6% and 2.0%, respectively.

Dai and collaborators [29] evaluated efficacy and safety outcomes of dabigatran-reversal with idarucizumab in *real-world* patients considering their eligibility for the REVERSE-AD trial. Interestingly, authors found that patients who would be eligible for the REVERSE-AD trial had higher successful haemostasis rates and anticoagulant effect reversal rates compared to those who would have been excluded from the study. Moreover, mortality rates were 9.5% in eligible patients compared to 27.3% in the ineligible group.

A retrospective observational case series on dabigatran users treated with idarucizumab was performed in Denmark [30]. The study showed a favourable benefit/risk profile, with 13% of subjects experiencing bleeding within 30 days after infusion and no thromboembolic complications. However, at 30 days of follow-up, 17% of patients died. Two patients died within two days and the others between five and 30 days after infusion.

In 2023, Singer and colleagues [20] conducted a multicenter chart review of adult patients with factor Xa-Inhibitor associated intracerebral haemorrhage or gastrointestinal bleeding treated with andexanet al.fa or factor-4 prothrombin complex concentrate (4 F-PCC). The rate of thrombotic events within 90 days from the administration was 14% among patients treated with andexanet al.fa of the drug and 16% in patients receiving 4 F-PCC. The survival rate to hospital discharge did not differ between the two groups, with rates of 92% for andexanet al.fa and 76% for 4 F-PCC patients (p-value 0.25).

In 2022, Chaudhary and colleagues published a systematic review and meta-analysis evaluating the safety and outcomes of DOAC reversal agents among patients with ICH [31]. Authors considered all-cause mortality and thromboembolic events after the reversal agent as primary safety outcomes. The analysis included 36 clinical studies for a total of 525 subjects treated with andexanet al.fa, and 340 subjects treated with idarucizumab. All-cause mortality rate for andexanet al.fa and idarucizumab accounted for 24% and 11%, respectively, while the rate for thromboembolic events accounted for 14% and 5%.

According to our results, the above-mentioned studies also confirm a prevalence of the male population in the study samples. However, unlike previous reviews and studies, our analysis explored the clinical characteristics potentially contributing to safety outcomes. No demographic characteristic increases the probability of severe ADRs or death. For example, there is no difference between genders for these outcomes. Previous pharmacovigilance studies showed that drug classes significantly associated with an increased risk of serious ADRs differed between the two sexes [32]. In our sample, the only clinical characteristic identified as a risk factor for serious ADR or death is the total number of administered drugs, both suspected/interacting and concomitant ones. As demonstrated by previous pharmacovigilance analyses [23, 25, 33], this evidence underlines the risk associated with drug-drug interactions (DDIs) that may be underestimated by clinicians and patients, especially in case of administration of medicinal products in an emergency setting or the administration of products belonging to the complementary and alternative medicines, such as phytotherapy and dietary supplements [26, 34]. According to a recent study, drugs administered to inpatients in addition to those already used for usual care may increase potential DDIs more than 3-fold [35] and DDIs may be identified in up to 80% of patients [36]. Moreover, in clinical practice, an increase in the number of medications a patient is taking correlates with a higher prevalence of underlying medical conditions and greater disease burden. This correlation underscores the concept of greater clinical complexity [37, 38]. These considerations are particularly pertinent in interpreting the results of our study, where the population predominantly comprises patients aged > 75 years. As reported in previous publications, older individuals represent a vulnerable population requiring particular attention. In fact, drug safety data for these populations are often limited and they are underrepresented in clinical trials [23]. However, as indicated by our results, in our sample, the probability of thromboembolic events is significantly lower in subjects aged more than 75 years. Notably, aging is associated with an increase in plasma levels of fibrinogen, high-molecular-weight kininogen, prekallikrein, factors V, VII, VIII, IX, XI, and von Willebrand factor, which have been shown to be risk factors for thrombotic disease. Moreover, venous wall structure, nitric oxide and endothelin-1 expression are impaired in this age group [39]. In addition to advancing age, other major acquired risk factors include current or recent hospitalization, malignancy, congestive heart failure, trauma, surgery, and chronic renal disease [40, 41]. However, conflicting evidence exists regarding age-related changes in the natural anticoagulants, including protein C, protein S, and antithrombin [42]. In fact, aging has also been associated with a decrease in the number of platelets, prostacyclin, and thromboxane A2 receptors, impairing platelet aggregation [43]. Moreover, changes in the level or activity of proteins within the fibrinolysis pathway, such as antithrombin, heparin cofactor II, and thrombin activatable fibrinolysis inhibitor (TAFI), may also potentially affect clot formation. In patients exposed to reversal agents, particularly andexanet al.fa, a pro-thrombotic rebound effect cannot be excluded as andexanet al.fa has shown to transiently increase thrombin generation [11]. However, published studies highlighted that older subjects showed a high haemostatic efficacy [44], suggesting that they may be less affected by the restoration of their baseline risk of thromboembolism after anticoagulation withdrawal due to the presence of a reversal agent, even when the restoration of anticoagulation therapy is incomplete and late [45]. However, observational pharmacovigilance studies could not delve into these aspects deeply, as the information contained within ICSRs is limited and rarely provides data concerning specific laboratory tests. On the other hand, older patients, especially those with comorbidities, are usually well-monitored, particularly in emergency conditions [23]. Therefore, it is possible that, given these patients are old, in an emergency condition, and undergoing treatment with drugs under additional monitoring, the reduced probability of thromboembolic events is due to intensive clinical monitoring. Another hypothesis to the limited number of thromboembolic events observed in patients aged more than 75 years is represented by the limited survival. In the event of death, regardless of whether it was caused by reversal agents, thromboembolic events may go unobserved and unreported. Moreover, information contained in ADR reports refer to a limited time frame, from the administration of the suspect agent and the occurrence of ADR up to few days after the event. Follow-up information is requested only in severe cases and rarely the observation period reaches thirty days or more [46]. Published studies analysed all-cause mortality and the rate of thromboembolic events within thirty days [44]. Therefore, this analysis could not identify thromboembolic events with a delayed onset. The prevalence of multiple medications and underlying health conditions among older subjects further accentuates the relevance of understanding and addressing clinical complexity in our findings.

### Limitations and strengths

There are several study limitations. The observational nature of pharmacovigilance may have led to an underestimation of ADRs, since underreporting is a well-known limitation of this type of analysis [47]. In fact, clinicians and healthcare givers may have not reported mild or moderate ADRs in favour of serious and fatal cases. Reasons for underreporting other than ignorance (the belief that only serious ADRs need to be reported) that may have led to an underestimation of our results are represented by procrastination and diffidence [48]. In particular, procrastination may be due to the emergency and critical conditions of patients, that may delay the reporting of ADRs, especially those classified as mild and moderate, through pharmacovigilance systems. Furthermore, our analysis is based on ADR reports that are affected by limits that include inaccurate and incomplete information, mainly due to the lack of demographic and clinical data. Among unavailable data, frailty of subjects included in this analysis was not assessable. According to the APULEIO study [49, 50] efficacy and safety outcomes may vary among patients treated with apixaban according to their clinical condition of disability, frailty and robustness. In light of this, safety outcomes during the use of andexanet al.fa may also depend on patient’s disability, frailty or robustness. Other lacking information is represented by results of post-mortem analysis that may clarify the cause of death and identify thrombotic events. Consequently, it was not always possible to exclude the absence of conditions related to clinical outcomes development. Since the data included in this analysis were extracted from a pharmacovigilance database that exclusively contains reports of patients who experienced an ADR, information on patients exposed to reversal agents without ADRs is unavailable. While the inclusion of a control group would have allowed for a more comprehensive evaluation of potential clinical and demographic risk factors for ADR occurrence, this study still provides valuable insights into the characteristics of patients experiencing ADRs, contributing to a better understanding of safety outcomes in real-world settings. Moreover, the evaluation of the latency of the ADRs was not possible, since not all pharmacovigilance ICSRs informed about the dates of administration and of ADR occurrence.

Despite these limitations, this is the first global analysis of ADRs associated with the use of idarucizumab and andexanet alfa in a real-world population. Pharmacovigilance assessments are crucial for monitoring drug safety, particularly in fields where expert opinions and small clinical studies prevail over large-scale epidemiological and clinical data collection efforts. This study contributes further evidence to recent pharmacovigilance research, identifying risk factors for ADRs seriousness and clinical outcomes associated with the use of these two DOACs reversal agents. These findings hold significant implications for clinicians, patients, policy-makers, and researchers.

## Conclusions

The findings of this study underscore the importance of pharmacovigilance monitoring and risk assessment when administering idarucizumab and andexanet alfa, particularly in older patients with concurrent medication regimens.

In real-world populations, ADRs associated with these drugs are mostly serious, and most serious reactions result in the death of the patient. Clinicians and healthcare providers must therefore pay particular attention when managing these drugs, especially in vulnerable individuals.

## Electronic supplementary material

Below is the link to the electronic supplementary material.

Supplementary Material 1

## Data Availability

The data that support the findings of this study are available from the corresponding author upon reasonable request.

## Declarations

### UMC statement

VigiBase, the WHO global database of ICSRs is the source of our information. The information comes from a variety of sources, and the probability that the suspected adverse event is drug-related is not the same in all cases; the information does not represent the opinion of the UMC or WHO.

## References

[1] Dabigatran versus warfarin in patients with atrial fibrillation - PubMed https://pubmed.ncbi.nlm.nih.gov/19717844/. Accessed 27 Jan 2025

[2] van der Hulle T, Kooiman J, den Exter PL et al (2014) Effectiveness and safety of novel oral anticoagulants as compared with vitamin K antagonists in the treatment of acute symptomatic venous thromboembolism: a systematic review and meta-analysis. J Thromb Haemost 12:320–328. 10.1111/jth.12485 24330006 10.1111/jth.12485

[3] Singh S, Nautiyal A, Belk KW (2020) Real world outcomes associated with Idarucizumab: Population-Based retrospective cohort study. Am J Cardiovasc Drugs 20:161–168. 10.1007/s40256-019-00360-6 31332727 10.1007/s40256-019-00360-6

[4] Bo M, Fumagalli S, Degli Esposti L et al (2024) Anticoagulation in atrial fibrillation. A large real-world update. Eur J Intern Med 121:88–94. 10.1016/j.ejim.2023.10.010 37879969 10.1016/j.ejim.2023.10.010

[5] Schulman S, Bijsterveld NR (2007) Anticoagulants and their reversal. Transfus Med Rev 21:37–48. 10.1016/j.tmrv.2006.08.002 17174219 10.1016/j.tmrv.2006.08.002

[6] van der Wall SJ, van Rein N, van den Bemt B et al (2019) Performance of Idarucizumab as antidote of Dabigatran in daily clinical practice. Europace 21:414–420. 10.1093/europace/euy220 30339226 10.1093/europace/euy220

[7] Schiele F, van Ryn J, Canada K et al (2013) A specific antidote for Dabigatran: functional and structural characterization. Blood 121:3554–3562. 10.1182/blood-2012-11-468207 23476049 10.1182/blood-2012-11-468207

[8] Garrett AD (2015) Idarucizumab for Dabigatran reversal. Drug Top 159:31

[9] van Ryn J, Stangier J, Haertter S et al (2010) Dabigatran etexilate–a novel, reversible, oral direct thrombin inhibitor: interpretation of coagulation assays and reversal of anticoagulant activity. Thromb Haemost 103:1116–1127. 10.1160/TH09-11-0758 20352166 10.1160/TH09-11-0758

[10] (2015) Praxbind| European Medicines Agency (EMA). https://www.ema.europa.eu/en/medicines/human/EPAR/praxbind. Accessed 27 Jan 2025

[11] (2019) Ondexxya| European Medicines Agency (EMA). https://www.ema.europa.eu/en/medicines/human/EPAR/ondexxya. Accessed 27 Jan 2025

[12] Al Aseri Z, AlGahtani FH, Bakheet MF et al (2023) Evidence-based management of major bleeding in patients receiving direct oral anticoagulants: an updated narrative review on the role of specific reversal agents. J Cardiovasc Pharmacol Ther 28:10742484231202655. 10.1177/10742484231202655 37872658 10.1177/10742484231202655

[13] Pollack CV, Reilly PA, van Ryn J et al (2017) Idarucizumab for Dabigatran Reversal - Full cohort analysis. N Engl J Med 377:431–441. 10.1056/NEJMoa1707278 28693366 10.1056/NEJMoa1707278

[14] Connolly SJ, Crowther M, Eikelboom JW et al (2019) Full study report of Andexanet Alfa for bleeding associated with factor Xa inhibitors. N Engl J Med 380:1326–1335. 10.1056/NEJMoa1814051 30730782 10.1056/NEJMoa1814051 PMC6699827

[15] Milling TJ, Middeldorp S, Xu L et al (2023) Final study report of Andexanet Alfa for major bleeding with factor Xa inhibitors. Circulation 147:1026–1038. 10.1161/CIRCULATIONAHA.121.057844 36802876 10.1161/CIRCULATIONAHA.121.057844

[16] Carpenter E, Singh D, Dietrich E, Gums J (2019) Andexanet Alfa for reversal of factor Xa inhibitor-associated anticoagulation. Ther Adv Drug Saf 10:2042098619888133. 10.1177/2042098619888133 31807265 10.1177/2042098619888133 PMC6880028

[17] Chodankar D (2021) Introduction to real-world evidence studies. Perspect Clin Res 12:171–174. 10.4103/picr.picr_62_21 34386383 10.4103/picr.picr_62_21 PMC8323556

[18] Lavertu A, Vora B, Giacomini KM et al (2021) A new era in pharmacovigilance: toward Real-World data and digital monitoring. Clin Pharmacol Ther 109:1197–1202. 10.1002/cpt.2172 33492663 10.1002/cpt.2172 PMC8058244

[19] Włodarczyk E, Sawczyńska K, Wrona P, Słowik A (2023) Reversing Dabigatran effect with Idarucizumab to enable intravenous thrombolysis in patients with acute ischaemic stroke - a single centre experience. Neurol Neurochir Pol 57:465–476. 10.5603/pjnns.96469 37955597 10.5603/pjnns.96469

[20] Singer AJ, Concha M, Williams J et al (2023) Treatment of factor-Xa Inhibitor-associated bleeding with Andexanet Alfa or 4 factor PCC: A multicenter feasibility retrospective study. West J Emerg Med 24:939–949. 10.5811/westjem.60587 37788035 10.5811/westjem.60587 PMC10527834

[21] Noseda R, Müller L, Bedussi F et al (2022) Immune checkpoint inhibitors and pregnancy: analysis of the VigiBase^®^ spontaneous reporting system. Cancers (Basel) 15:173. 10.3390/cancers15010173 36612168 10.3390/cancers15010173 PMC9818632

[22] Lombardi N, Bettiol A, Crescioli G et al (2020) Risk of hospitalisation associated with benzodiazepines and z-drugs in Italy: a nationwide multicentre study in emergency departments. Intern Emerg Med 15:1291–1302. 10.1007/s11739-020-02339-7 32333265 10.1007/s11739-020-02339-7

[23] Crescioli G, Bettiol A, Bonaiuti R et al (2020) Risk of hospitalization associated with cardiovascular medications in the elderly Italian population: A nationwide multicenter study in emergency departments. Front Pharmacol 11:611102. 10.3389/fphar.2020.611102 33708120 10.3389/fphar.2020.611102 PMC7941274

[24] Pagani S, Lombardi N, Crescioli G et al (2022) Drug-Related hypersensitivity reactions leading to emergency department: original data and systematic review. J Clin Med 11:2811. 10.3390/jcm11102811 35628936 10.3390/jcm11102811 PMC9143688

[25] Lombardi N, Crescioli G, Bettiol A et al (2020) Italian emergency department visits and hospitalizations for outpatients’ adverse drug events: 12-Year active pharmacovigilance surveillance (The MEREAFaPS Study). Front Pharmacol 11:412. 10.3389/fphar.2020.00412 32327995 10.3389/fphar.2020.00412 PMC7153477

[26] Lombardi N, Crescioli G, Maggini V et al (2022) Adverse events related to herbal dietary supplements and over-the-counter medications containing laxatives: a 10-year update from the Italian phytovigilance and pharmacovigilance systems. Ann Ist Super Sanita 58:131–138. 10.4415/ANN_22_02_09 35722800 10.4415/ANN_22_02_09

[27] Romoli M, Matteo E, Migliaccio L et al (2023) Thrombolysis after Dabigatran reversal: A nation-wide Italian multicentre study, systematic review and meta-analysis. Eur Stroke J 8:117–124. 10.1177/23969873221131635 37021155 10.1177/23969873221131635 PMC10069212

[28] van der Horst SFB, Martens ESL, den Exter PL et al (2023) Idarucizumab for Dabigatran reversal: A systematic review and meta-analysis of indications and outcomes. Thromb Res 228:21–32. 10.1016/j.thromres.2023.05.020 37267671 10.1016/j.thromres.2023.05.020

[29] Dai J-W, Wang C-H, Chu C-L, Liao S-C (2023) Effectiveness and safety of Dabigatran reversal with Idarucizumab in the Taiwanese population: A comparison based on eligibility for inclusion in clinical trials. Med (Kaunas) 59:881. 10.3390/medicina59050881 10.3390/medicina59050881 PMC10224188 37241113

[30] Haastrup SB, Hellfritzsch M, Nybo M et al (2021) Real-world experience with reversal of Dabigatran by Idarucizumab. Thromb Res 197:179–184. 10.1016/j.thromres.2020.11.010 33227654 10.1016/j.thromres.2020.11.010

[31] Chaudhary R, Singh A, Chaudhary R et al (2022) Evaluation of direct oral anticoagulant reversal agents in intracranial hemorrhage: A systematic review and Meta-analysis. JAMA Netw Open 5:e2240145. 10.1001/jamanetworkopen.2022.40145 36331504 10.1001/jamanetworkopen.2022.40145 PMC9636520

[32] Crescioli G, Boscia E, Bettiol A et al (2021) Risk of hospitalization for adverse drug events in women and men: A post hoc analysis of an active pharmacovigilance study in Italian emergency departments. Pharmaceuticals (Basel) 14:678. 10.3390/ph14070678 34358104 10.3390/ph14070678 PMC8308702

[33] Crescioli G, Brilli V, Lanzi C et al (2021) Adverse drug reactions in SARS-CoV-2 hospitalised patients: a case-series with a focus on drug-drug interactions. Intern Emerg Med 16:697–710. 10.1007/s11739-020-02586-8 33355896 10.1007/s11739-020-02586-8 PMC7755981

[34] Crescioli G, Maggini V, Raschi E et al (2023) Suspected adverse reactions to medications and food supplements containing Serenoa Repens: A worldwide analysis of pharmacovigilance and phytovigilance spontaneous reports. Phytother Res 37:5289–5299. 10.1002/ptr.7960 37463655 10.1002/ptr.7960

[35] Bischof T, Nagele F, Kalkofen MM et al (2024) Drug-drug-interactions in patients with atrial fibrillation admitted to the emergency department. Front Pharmacol 15:1432713. 10.3389/fphar.2024.1432713 39508037 10.3389/fphar.2024.1432713 PMC11538323

[36] Okuno MFP, Cintra RS, Vancini-Campanharo CR, Batista REA (2013) Drug interaction in the emergency service. Einstein (Sao Paulo) 11:462–466. 10.1590/s1679-45082013000400010 24488385 10.1590/S1679-45082013000400010 PMC4880383

[37] Vocca C, Siniscalchi A, Rania V et al (2023) The risk of drug interactions in older primary care patients after hospital discharge: the role of drug reconciliation. Geriatr (Basel) 8:122. 10.3390/geriatrics8060122 10.3390/geriatrics8060122 PMC10742527 38132493

[38] Charlson ME, Pompei P, Ales KL, MacKenzie CR (1987) A new method of classifying prognostic comorbidity in longitudinal studies: development and validation. J Chronic Dis 40:373–383. 10.1016/0021-9681(87)90171-8 3558716 10.1016/0021-9681(87)90171-8

[39] Akrivou D, Perlepe G, Kirgou P et al (2022) Pathophysiological aspects of aging in venous thromboembolism: an update. Med (Kaunas) 58:1078. 10.3390/medicina58081078 10.3390/medicina58081078 PMC9415158 36013544

[40] Anderson FA, Spencer FA (2003) Risk factors for venous thromboembolism. Circulation 107:I9–16. 10.1161/01.CIR.0000078469.07362.E6 12814980 10.1161/01.CIR.0000078469.07362.E6

[41] Tagalakis V, Patenaude V, Kahn SR, Suissa S (2013) Incidence of and mortality from venous thromboembolism in a real-world population: the Q-VTE study cohort. Am J Med 126:832e13–832e21. 10.1016/j.amjmed.2013.02.024 10.1016/j.amjmed.2013.02.024 23830539

[42] Favaloro EJ, Franchini M, Lippi G (2014) Aging hemostasis: changes to laboratory markers of hemostasis as we age - a narrative review. Semin Thromb Hemost 40:621–633. 10.1055/s-0034-1384631 25099191 10.1055/s-0034-1384631

[43] Abbate R, Prisco D, Rostagno C et al (1993) Age-related changes in the hemostatic system. Int J Clin Lab Res 23:1–3. 10.1007/BF02592271 8477086 10.1007/BF02592271

[44] Connolly SJ, Sharma M, Cohen AT et al (2024) Andexanet for factor Xa Inhibitor-Associated acute intracerebral hemorrhage. N Engl J Med 390:1745–1755. 10.1056/NEJMoa2313040 38749032 10.1056/NEJMoa2313040

[45] Gómez-Outes A, Alcubilla P, Calvo-Rojas G et al (2021) Meta-Analysis of reversal agents for severe bleeding associated with direct oral anticoagulants. J Am Coll Cardiol 77:2987–3001. 10.1016/j.jacc.2021.04.061 34140101 10.1016/j.jacc.2021.04.061

[46] Vemula GK, Badale P, Mavrogenis P et al (2024) A Risk-Based approach for safety case Follow-up of adverse event reports in pharmacovigilance. Adv Ther 41:82–91. 10.1007/s12325-023-02699-4 37919600 10.1007/s12325-023-02699-4 PMC10796573

[47] Güner MD, Ekmekci PE (2019) Healthcare professionals’ pharmacovigilance knowledge and adverse drug reaction reporting behavior and factors determining the reporting rates. J Drug Assess 8:13–20. 10.1080/21556660.2019.1566137 30729064 10.1080/21556660.2019.1566137 PMC6352929

[48] García-Abeijon P, Costa C, Taracido M et al (2023) Factors associated with underreporting of adverse drug reactions by health care professionals: A systematic review update. Drug Saf 46:625–636. 10.1007/s40264-023-01302-7 37277678 10.1007/s40264-023-01302-7 PMC10279571

[49] Fumagalli S, Di Pasquale G, Pupo S et al (2024) Patient-reported outcomes and Apixaban therapy in older patients. Eur J Intern Med 124:156–159. 10.1016/j.ejim.2024.02.034 38472046 10.1016/j.ejim.2024.02.034

[50] Fumagalli S, Di Pasquale G, Molon G et al (2025) Frailty, disability and patient-reported outcomes in Apixaban users. Eur J Intern Med S. 10.1016/j.ejim.2025.01.022. 0953-6205(25)00032–9 10.1016/j.ejim.2025.01.022 39890567

