# Supplementary material for: Safety of direct oral anticoagulants reversal agents in older patients: an analysis of individual case safety reports of adverse drug reaction from VigiBase®
Source: Aging Clin Exp Res. 2025 Apr 7;37(1):120. doi: 10.1007/s40520-025-03025-4 (PMC11976745; doi:10.1007/s40520-025-03025-4)
Supplement: Supplementary file 1 — Supplementary Material 1 [file 40520_2025_3025_MOESM1_ESM.docx]

**Supplementary file 1**

**Supplementary Table 1.** Most frequently reported preferred terms

| PT | N | % out of 2180 |
| --- | --- | --- |
| Death | 159 | 7,29 |
| Haemorrhage | 74 | 3,39 |
| Off label use | 57 | 2,61 |
| Cerebral haemorrhage | 55 | 2,52 |
| Gastrointestinal haemorrhage | 50 | 2,29 |
| Cerebrovascular accident | 40 | 1,83 |
| Haemorrhage intracranial | 40 | 1,83 |
| Drug ineffective | 39 | 1,79 |
| Ischaemic stroke | 34 | 1,56 |
| Cerebral infarction | 30 | 1,38 |
| Multiple organ dysfunction syndrome | 30 | 1,38 |
| Acute myocardial infarction | 29 | 1,33 |
| Pulmonary embolism | 27 | 1,24 |
| Sepsis | 27 | 1,24 |
| Pneumonia | 24 | 1,10 |
| Hypotension | 21 | 0,96 |
| Acute kidney injury | 21 | 0,96 |
| General physical health deterioration | 17 | 0,78 |
| Myocardial infarction | 17 | 0,78 |
| Septic shock | 17 | 0,78 |
| Coagulopathy | 16 | 0,73 |
| Deep vein thrombosis | 16 | 0,73 |
| Cardiac arrest | 15 | 0,69 |
| Haemoglobin decreased | 15 | 0,69 |
| Renal failure | 15 | 0,69 |
| Thrombosis | 14 | 0,64 |
| Subdural haematoma | 14 | 0,64 |
| Shock haemorrhagic | 14 | 0,64 |
| Melaena | 14 | 0,64 |
| Cardiac failure | 14 | 0,64 |
| Activated partial thromboplastin time prolonged | 13 | 0,60 |
| Rectal haemorrhage | 12 | 0,55 |
| Drug interaction | 11 | 0,50 |
| Procedural haemorrhage | 11 | 0,50 |
| Respiratory failure | 11 | 0,50 |
| Infection | 11 | 0,50 |
| Shock | 11 | 0,50 |
| Embolism | 10 | 0,46 |
| Fall | 10 | 0,46 |
| Product temperature excursion issue | 10 | 0,46 |
| Haematemesis | 10 | 0,46 |

**Supplementary Table 2.** Concomitant medications

| **Medication** | **N=2387 (%)** |
| --- | --- |
| Furosemide | 79 (3.31) |
| Apixaban | 78 (3.27) |
| Bisoprolol | 72 (3.02) |
| Atorvastatin | 58 (2.43) |
| Rivaroxaban | 52 (2.18) |
| Pantoprazole | 50 (2.09) |
| Metoprolol | 48 (2.01) |
| Amlodipine | 46 (1.93) |
| Dabigatran | 46 (1.93) |
| Omeprazole | 39 (1.63) |
| Spironolactone | 38 (1.59) |
| Acetylsalicylic acid | 37 (1.55) |
| Levothyroxine | 34 (1.42) |
| Insulin | 30 (1.26) |
| Magnesium | 29 (1.21) |
| Paracetamol | 28 (1.17) |
| Allopurinol | 25 (1.05) |
| Carvedilol | 24 (1.01) |
| Digoxin | 24 (1.01) |
| Ramipril | 24 (1.01) |
| Torasemide | 23 (0.96) |
| Amiodarone | 22 (0.92) |
| Potassium salts | 22 (0.92) |
| Rosuvastatin | 21 (0.88) |
| Metformin | 20 (0.84) |
| Simvastatin | 19 (0.80) |
| Multivitamin complex | 18 (0.75) |
| Norepinephrine | 17 (0.71) |
| Colecalciferol | 15 (0.63) |
| Diltiazem | 15 (0.63) |
| Telmisartan | 15 (0.63) |
| Candesartan | 14 (0.59) |
| Clopidogrel | 14 (0.59) |
| Folic acid | 14 (0.59) |
| Heparin | 14 (0.59) |
| Edoxaban | 13 (0.54) |
| Esomeprazole | 13 (0.54) |
| Lansoprazole | 13 (0.54) |
| Sodium | 13 (0.54) |
| Lisinopril | 12 (0.50) |
| Metamizole | 12 (0.50) |
| Perindopril | 12 (0.50) |
| Fentanyl | 11 (0.46) |
| Nicardipine | 11 (0.46) |
| Calcium salts | 10 (0.42) |
| Isosorbide | 10 (0.42) |
| Losartan | 10 (0.42) |
| Tranexamic acid | 10 (0.42) |
